# Scrotal Calculus

**Published:** 1882-03

**Authors:** 


### SCROTAL CALCULUS.

A unique case of this affection is reported by Lippman. The patient, a peasant, sixty-eight years old, stated that fifteen years previously he had suffered from difficulty in passing water, which finally became so aggravated that he consulted a physician, who removed from the scrotum by incision a stone weighing one hundred and twenty grammes. An urethral fistula resulted, through which all the urine passed, at first with ease, but of late with increasing difficulty. On examination a fistula was found just behind the peno-scrotal junction All endeavors to pass a metallic or an elastic catheter per urethram were futile; only the anterior portion of the pendulous urethra was permeable. The scrotum was as large as a child's head, and was distended by a hard mass. The testicles were dislocated toward the external inguinal apertures, and were very much atrophied. On introducing a catheter into the fistula, it was arrested by a stone. On the following day an incision was carried from the fistula backward along the raphe of the scrotum, and four calculi, weighing in all forty grammes were removed. They were rough, of a grayish-yellow color, showed facets, and consisted of phosphates. When placed in apposition they were as large as a goose egg. The cavity left after the operation was cleansed with carbolic acid, drained, and healed kindly, leaving a fistula, through which the patient urinated. Later he emptied his bladder with the aid of a self-made catheter consisting of a goose-quill.—*Centralblatt fuer Chirurgie.*

